# Supplementary material for: Psittacid Adenovirus-2 infection in the critically endangered orange-bellied parrot (Neophema chrysogastor): A key threatening process or an example of a host-adapted virus?
Source: PLoS One. 2019 Feb 27;14(2):e0208674. doi: 10.1371/journal.pone.0208674 (PMC6392234; doi:10.1371/journal.pone.0208674)
Supplement: S1 File — (DOCX) [file pone.0208674.s001.docx]

**Supporting Information File 1: Psittacid adenovirus-2 infection status, sample type, age, sex and origin of orange-bellied parrots used in this study.**

| **Infection Status** | **Sample Type** | **Age** | **Sex** | **Origin** |
| --- | --- | --- | --- | --- |
| + | Swab | ≥1 | Female | Werribee Zoo |
| - | Swab | ≥1 | Female |  |
| - | Swab | ≥1 | Female |  |
| - | Swab | ≥1 | Female |  |
| - | Swab | ≥1 | Female |  |
| + | Swab | ≥1 | Female |  |
| + | Swab | ≥1 | Female |  |
| + | Swab | ≥1 | Female |  |
| + | Swab | ≥1 | Male | **Priam** |
| + | Swab | ≥1 | Female |  |
| - | Swab | ≥1 | Male |  |
| + | Swab | ≥1 | Male |  |
| + | Swab | ≥1 | Male |  |
| + | Swab | ≥1 | Male |  |
| + | Swab | ≥1 | Female |  |
| + | Swab | ≥1 | Male |  |
| + | Swab | ≥1 | Female |  |
| + | Swab | ≥1 | Male |  |
| + | Swab | ≥1 | Male |  |
| - | Swab | ≥1 | Male |  |
| + | Swab | ≥1 | Female |  |
| + | Swab | ≥1 | Female |  |
| - | Swab | <1 | Male |  |
| - | Swab | <1 | Male |  |
| + | Swab | <1 | Female |  |
| + | Swab | ≥1 | Male | **Moonlit** |
| + | Swab | <1 | Male |  |
| - | Swab | <1 | Male |  |
| - | Swab | ≥1 | Male |  |
| - | Swab | <1 | Female |  |
| - | Swab | ≥1 | Male |  |
| - | Swab | ≥1 | Female |  |
| + | Swab | ≥1 | Male |  |
| + | Swab | ≥1 | Female |  |
| - | Swab | <1 | Female |  |
| + | Swab | ≥1 | Male |  |
| + | Swab | ≥1 | Female |  |
| - | Swab | <1 | Female |  |
| + | Swab | ≥1 | Female |  |
| - | Swab | <1 | Male |  |
| + | Swab | ≥1 | Male |  |
| - | Swab | <1 | Female |  |
| - | Swab | <1 | Male |  |
| - | Swab | ≥1 | Female |  |
| + | Swab | ≥1 | Female |  |
| - | Swab | <1 | Female |  |
| - | Swab | <1 | Male |  |
| - | Swab | <1 | Female |  |
| - | Swab | <1 | Male |  |
| + | Swab | ≥1 | Male |  |
| + | Swab | ≥1 | Male | **Adelaide Zoo** |
| + | Swab | ≥1 | Male |  |
| - | Swab | ≥1 | Female |  |
| - | Swab | ≥1 | Male |  |
| + | Swab | ≥1 | Male |  |
| + | Swab | ≥1 | Male |  |
| + | Swab | ≥1 | Female |  |
| + | Swab | ≥1 | Male |  |
| - | Swab | ≥1 | Female |  |
| - | Swab | ≥1 | Male |  |
| - | Swab | ≥1 | Female |  |
| - | Swab | ≥1 | Male |  |
| + | Swab | ≥1 | Male |  |
| - | Swab | ≥1 | Female |  |
| + | Swab | ≥1 | Female |  |
| - | Swab | ≥1 | Female |  |
| + | Swab | ≥1 | Female |  |
| - | Swab | <1 | Female |  |
| - | Swab | <1 | Female |  |
| - | Swab | <1 | Female |  |
| - | Swab | <1 | Female |  |
| - | Swab | <1 | Female |  |
| - | Swab | <1 | Male |  |
| - | Swab | Not provided | Not provided | Healesville |
| + | Swab | Not provided | Not provided |  |
| - | Swab | Not provided | Not provided |  |
| - | Swab | Not provided | Not provided |  |
| - | Swab | Not provided | Not provided |  |
| - | Swab | Not provided | Not provided |  |
|  | Swab | - | Not provided |  |
| - | Swab | Not provided | Not provided |  |
| - | Swab | Not provided | Not provided |  |
| + | Swab | Not provided | Not provided |  |
| + | Swab | Not provided | Not provided |  |
| - | Swab | Not provided | Not provided |  |
| - | Swab | Not provided | Not provided |  |
| - | Swab | Not provided | Not provided |  |
| - | Swab | Not provided | Not provided |  |
| - | Swab | Not provided | Not provided |  |
| - | Swab | Not provided | Not provided |  |
| - | Swab | Not provided | Not provided |  |
| - | Swab | Not provided | Not provided |  |
| - | Swab | Not provided | Not provided |  |
| - | Swab | Not provided | Not provided |  |
| + | Swab | Not provided | Not provided |  |
| - | Swab | Not provided | Not provided |  |
| - | Swab | Not provided | Not provided |  |
| - | Swab | Not provided | Not provided |  |
| - | Swab | Not provided | Not provided |  |
| - | Swab | Not provided | Not provided |  |
| - | Swab | Not provided | Not provided |  |
| - | Swab | Not provided | Not provided |  |
| - | Swab | Not provided | Not provided |  |
| + | Swab | Not provided | Not provided |  |
| - | Swab | Not provided | Not provided |  |
| + | Swab | Not provided | Not provided |  |
| + | Swab | Not provided | Not provided |  |
| - | Swab | Not provided | Not provided |  |
| - | Swab | Not provided | Not provided |  |
| + | Swab | Not provided | Not provided |  |
| - | Fecal | Not provided | Not provided |  |
| + | Fecal | Not provided | Not provided |  |
| - | Fecal | Not provided | Not provided |  |
| - | Fecal | Not provided | Not provided |  |
| - | Fecal | Not provided | Not provided |  |
| - | Fecal | Not provided | Not provided |  |
| - | Fecal | Not provided | Not provided |  |
| - | Fecal | Not provided | Not provided |  |
| - | Fecal | Not provided | Not provided |  |
| + | Fecal | Not provided | Not provided |  |
| + | Fecal | Not provided | Not provided |  |
| - | Fecal | Not provided | Not provided |  |
| - | Fecal | Not provided | Not provided |  |
| - | Fecal | Not provided | Not provided |  |
| - | Fecal | Not provided | Not provided |  |
| - | Fecal | Not provided | Not provided |  |
| - | Fecal | Not provided | Not provided |  |
| - | Fecal | Not provided | Not provided |  |
| - | Fecal | Not provided | Not provided |  |
| - | Fecal | Not provided | Not provided |  |
| - | Fecal | Not provided | Not provided |  |
| + | Fecal | Not provided | Not provided |  |
| - | Fecal | Not provided | Not provided |  |
| - | Fecal | Not provided | Not provided |  |
| + | Fecal | Not provided | Not provided |  |
| + | Fecal | Not provided | Not provided |  |
| + | Fecal | Not provided | Not provided |  |
| - | Fecal | Not provided | Not provided |  |
| - | Fecal | Not provided | Not provided |  |
| + | Fecal | Not provided | Not provided |  |
| - | Fecal | Not provided | Not provided |  |
| + | Fecal | Not provided | Not provided |  |
| + | Fecal | Not provided | Not provided |  |
| - | Fecal | Not provided | Not provided |  |
| - | Fecal | Not provided | Not provided |  |
