# Supplementary material for: Psittacid Adenovirus-2 infection in the critically endangered orange-bellied parrot (Neophema chrysogastor): A key threatening process or an example of a host-adapted virus?
Source: PLoS One. 2019 Feb 27;14(2):e0208674. doi: 10.1371/journal.pone.0208674 (PMC6392234; doi:10.1371/journal.pone.0208674)
Supplement: S2 File — (DOCX) [file pone.0208674.s002.docx]

**Supporting Information File 2: Distribution of reproductive data and infection prevalence in four breeding colonies of the orange-bellied parrot.**

|  | **Fertility Rate** | **Hatch Rate(of fertile eggs)** | **Fledgling Rate(of chicks hatched)** | **PsAdv2 Prevalence** |
| --- | --- | --- | --- | --- |
| **Healesville Sanctuary** | 52% | 85% | 93% | 29.7% |
| **Adelaide Zoo** | 61% | 88% | 67% | 39% |
| **Moonlit Sanctuary** | 48% | 96% | 85% | 40% |
| **Priam Parrot Breeding Centre** | 38% | 100% | 37% | 76.5% |
